# Usefulness of endoscopic ultrasound‐guided transhepatic biliary drainage with a 22‐gauge fine‐needle aspiration needle and 0.018‐inch guidewire in the procedure's induction phase

**DOI:** 10.1002/deo2.297

**Published:** 2023-10-10

**Authors:** Kei Yane, Masahiro Yoshida, Takayuki Imagawa, Kotaro Morita, Hideyuki Ihara, Kota Hanada, Sota Hirokawa, Yusuke Tomita, Takeyoshi Minagawa, Yutaka Okagawa, Tetsuya Sumiyoshi, Michiaki Hirayama, Hitoshi Kondo

**Affiliations:** ^1^ Department of Gastroenterology Tonan Hospital Sapporo Japan

**Keywords:** biliary stricture, biliary drainage, endoscopic ultrasonography, EUS‐guided hepaticogastrostomy, EUS‐guided hepaticojejunostomy

## Abstract

Endoscopic ultrasound (EUS)‐guided transhepatic biliary drainage is usually performed with a 19‐gauge fine‐needle aspiration (FNA) needle and a 0.025‐inch guidewire. The combination of a 22‐gauge FNA needle and a 0.018‐inch guidewire is reported to be effective as a rescue option when the bile duct diameter is small or technically challenging. Experts in EUS‐guided transhepatic biliary drainage have reported that bile duct puncture with a 19‐gauge FNA needle is possible in most cases, but is not easy to reproduce by endoscopists with less experience in EUS‐guided transhepatic biliary drainage. We investigated the usefulness of EUS‐guided transhepatic biliary drainage using a 22‐gauge FNA needle and a 0.018‐inch guidewire during the procedure's induction phase. Consecutive patients who underwent EUS‐guided transhepatic biliary drainage at our institution from March 2021 to May 2023 were evaluated, and 37 were included. Biliary drainage was performed for malignant bile duct stricture in 36 patients and choledocholithiasis in one patient. The median target bile duct diameter was 4.5 mm (2.5–9.4). Biliary access, fistula dilation, and stent placement were successful in the 37 patients (100%). The median procedure time was 35 min (16–125). Adverse events occurred in four (10.8%) patients. EUS‐guided transhepatic biliary drainage using a 22‐gauge FNA needle and a 0.018‐inch guidewire is a useful and promising option for endoscopists with limited experience in EUS‐guided transhepatic biliary drainage in the procedure's induction phase.

## INTRODUCTION

Recently, endoscopic ultrasound (EUS)‐guided biliary drainage (EUS‐BD) has become a widely used method of bile duct drainage for obstructive jaundice.^1^, ^2^ EUS‐BD can be categorized into EUS‐guided choledochoduodenostomy (EUS‐CDS) and EUS‐guided transhepatic biliary drainage, depending on the approach route. EUS‐guided transhepatic biliary drainage is the treatment of choice when conventional endoscopic retrograde cholangiopancreatography (ERCP) is difficult because of duodenal obstruction or surgically altered anatomy.^3^, ^4^ In some cases, antegrade stenting or bridging drainage to the right liver lobe is performed simultaneously.^5^, ^6^

Regarding EUS‐guided transhepatic biliary drainage, many studies have reported high procedural success rates.^7^, ^8^, ^9^ However, most reports are from high‐volume centers, and EUS‐guided transhepatic biliary drainage is not an easy procedure for endoscopists with little experience, particularly during the procedure's induction phase. The technical success rate during the induction phase of EUS‐guided transhepatic biliary drainage is relatively low at 64.7%.^10^ A number of cases is required to stabilize the technique.^11^ Therefore, various technical tips have been reported to improve the success rate.^12^ Puncture of the target bile duct, cholangiography, and placement of a guidewire into the bile duct are very important early steps in EUS‐guided transhepatic biliary drainage. Although the combination of a 19‐gauge fine‐needle aspiration (FNA) needle and a 0.025‐inch guidewire is commonly used for these steps owing to good guidewire maneuverability and compatibility with dilation devices,^13^ in some cases, it is difficult to find an adequate puncture line with a 19‐gauge FNA needle owing to thin bile ducts or intervening blood vessels. In such cases, the usefulness of a 22‐gauge FNA needle has been reported.^9^, ^14^ In addition to improved puncture ability, a 22‐gauge FNA needle provides a wider range of needle movement than a 19‐gauge FNA needle, making it easier to select the bile duct branch that can be punctured.^12^

However, a 0.018‐inch guidewire used previously is not the first method of choice because of its inferior maneuverability compared with a 0.025‐inch guidewire and the limited dilation devices available after guidewire placement.^13^ Therefore, EUS‐guided transhepatic biliary drainage using a 22‐gauge FNA needle and a 0.018‐inch guidewire was reported as a rescue option when the bile duct diameter was small and difficult to puncture.

Recently, a 0.018‐inch guidewire with improved maneuverability has become commercially available and compatible with dilation devices.^15^, ^16^ Although the usefulness of EUS‐guided transhepatic biliary drainage with this new 0.018‐inch guidewire has also been reported, the results are mainly for difficult cases in high‐volume centers where EUS‐guided transhepatic biliary drainage is performed in large numbers.^17^ For this method to be commonly used, it was necessary to study the results in non‐high‐volume centers among endoscopists with little experience in performing EUS‐guided transhepatic biliary drainage.

Therefore, a retrospective observational study was conducted to investigate the usefulness of EUS‐guided transhepatic biliary drainage with a 22‐gauge FNA needle and a 0.018‐inch guidewire in the induction phase of EUS‐guided transhepatic biliary drainage in a non‐high‐volume center.

## PROCEDURE

### Study design

This retrospective observational study was conducted at Tonan Hospital, Sapporo, Hokkaido, Japan. The study protocol was approved by our hospital's ethics committee (institutional ID: 2022‐1‐8‐1). All participants provided written informed consent before the procedure.

### Patients

Consecutive patients who underwent EUS‐guided transhepatic biliary drainage at our institution from March 2021 to May 2023 were evaluated. Age, sex, indications of EUS‐guided transhepatic biliary drainage, site of bile duct stenosis, puncture site, diameter of the targeted bile duct, success rate of biliary access, technical success rate, clinical success rate, total procedure time, and adverse events rate were analyzed. Data associated with the endoscopic procedure were evaluated using the interventional EUS database, endoscopy reports, and video records.

### Definitions

*Success* of biliary access was defined as successful bile duct puncture and insertion of a guidewire into the bile duct.

*Technical success* was defined as the successful placement of a stent in the appropriate position.

*Clinical success* was defined as a 50% decrease in or normalization of the serum total bilirubin level in jaundice cases. In cases with segmental cholangitis, the disappearance of clinical symptoms including abdominal pain and fever was considered a clinical success.

*Total procedure time* was defined as the time from scope insertion to removal.

*Adverse events* were classified and graded according to the American Society for Gastrointestinal Endoscopy lexicon.^18^

### EUS‐guided transhepatic biliary drainage

**FIGURE 1:**
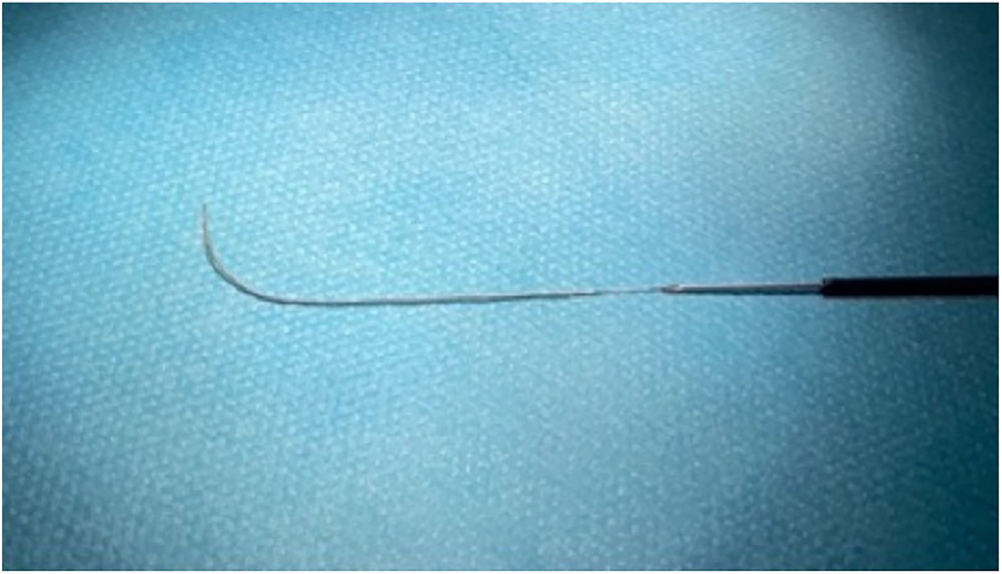
A 0.018‐inch guidewire (Fielder 18; Olympus) inserted into a 22‐gauge fine‐needle aspiration needle (Expect Slimline; Boston Scientific).

All endoscopic procedures were performed or supervised by a physician (Kei Yane) who has experience with more than 2000 ERCPs and 500 EUS‐FNA procedures, but less than 10 EUS‐guided transhepatic biliary drainage experience at the time of the first procedure of this study (Video S1). Two other endoscopists (Masahiro Yoshida: two cases of EUS‐guided transhepatic biliary drainage before the study; Takayuki Imagawa: no EUS‐guided transhepatic biliary drainage experience) also performed the procedure during the study period. As regards EUS‐guided interventions other than transhepatic biliary drainage, Kei Yane has an experience of 22 cases of cyst drainage, three cases of pancreatic duct drainage, three cases of gallbladder drainage (GBD), and two cases of CDS; Masahiro Yoshida has an experience of one case of GBD; Takayuki Imagawa has no experience with any of these. There were four assistants (Kei Yane, Masahiro Yoshida, Kotaro Morita, and Hideyuki Ihara: overlapping with the endoscopists), two with less than 10 cases of EUS‐guided transhepatic biliary drainage assistance, and two with no experience in EUS‐guided transhepatic biliary drainage assistance. A 22‐gauge FNA needle (EXPECT Slimline; Boston Scientific) and a 0.018‐inch guidewire (Fielder 18; Olympus Medical Systems) were used with an oblique‐viewing linear echoendoscope (EG‐580UT or EG‐740UT; Fujifilm Corp.) for all cases (Figure 1). First, an endoscopic clip (SureClip; Micro‐tech) was placed at the esophagogastric junction to provide a guide on the fluoroscopic image. Subsequently, the target bile duct (B2 or B3) was visualized, and the bile duct diameter at the puncture site was measured and punctured with a 22‐gauge FNA needle. Following cholangiography, a 0.018‐inch guidewire was advanced and placed in the bile duct through the FNA needle.

**FIGURE 2:**
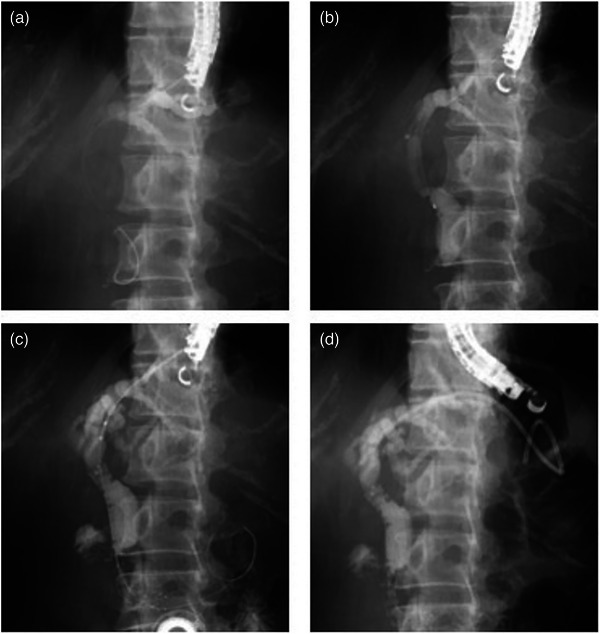
(a) Following cholangiography, a 0.018‐inch guidewire was advanced and placed in the bile duct through a 22‐gauge FNA needle. (b) An ERCP catheter was inserted into the bile duct, bile was aspirated to decompress the bile duct, and cholangiography was performed to confirm the stenosis. (c) A laser‐cut type uncovered metal stent was deployed across the papilla. (d) A plastic stent was deployed from the intrahepatic bile duct to the jejunum.

After the guidewire was inserted and advanced into the hilar side of the biliary tree, tract dilation was performed as required. A tapered tip ERCP catheter (ERCP CATHETER; MTW Endoskopie), a double‐lumen catheter (Uneven double lumen cannula; Piolax), tapered tip mechanical dilator (ES dilator; Zeon Medical), balloon dilator (REN; Kaneka), and drill dilator (Tornus ES; Olympus Medical Systems) were used for tract dilation at each endoscopist's discretion. An ERCP catheter was then inserted into the bile duct. Bile was aspirated to decompress the bile duct, and cholangiography was performed to confirm the stenosis. A 0.025‐inch guidewire (VisiGlide 2; Olympus Medical Systems and EndoSelector; Boston Scientific) was then inserted and placed in the appropriate position (i.e., common bile duct, intrahepatic bile duct, duodenum, or jejunum) depending on the case and the planned procedure, and a biliary stent was placed (Figure 2).

As a principle, in cases where antegrade stenting was planned in addition to EUS‐guided transhepatic biliary drainage, a laser‐cut type uncovered metal stent with a thin delivery system (ZEOSTENT V; Zeon Medical) was used, and a plastic stent (TYPE‐IT; Gadelius Medical) was used in the fistula site. In cases with ascites in the puncture tract, partially covered metal stents (Niti‐S S‐type or Spring Stopper; Taewoong Medical Inc.) were used at the fistula site.

In cases where only EUS‐guided transhepatic biliary drainage was planned, partially covered metal stents or plastic stents were used at the endoscopist's discretion. Clinical symptoms and physical findings were recorded after the procedure. Laboratory data and computed tomography were also assessed the following day. If there were no adverse events, the patient was allowed to eat the following day.

## RESULTS

Thirty‐seven patients (median age, 71 years; 21 men and 16 women) were included. The patients’ characteristics are shown in Table 1. The reason for biliary drainage was malignant bile duct stricture in 36 patients (pancreatic cancer, 17; gastric cancer, 10; gallbladder cancer, two; cholangiocarcinoma, two; other malignancy, five) and choledocholithiasis in one patient. The indications of EUS‐guided transhepatic biliary drainage were surgically altered anatomy (*n* = 20), duodenal obstruction (*n* = 10), segmental cholangitis that is difficult to control by ERCP (*n* = 5), and obscure ampulla due to tumor invasion (*n* = 2). The sites of bile duct stenosis were distal (*n* = 27), hilar (*n* = 6), choledochojejunal anastomosis (*n* = 2), and distal plus hilar (*n* = 1). There was no bile duct stenosis in the choledocholithiasis patient. The puncture target was B3 in 23 patients (62.2%) and B2 in 14 patients (37.8%). The puncture site was the stomach in 30 patients (81.1%) and the jejunum in seven patients (18.9%). The median target bile duct diameter was 4.5 mm (2.5–9.4).

**TABLE 1:** Patient characteristics.

Age, median (range), years	71 (40–88)
Sex (male/female)	21/16
Disease, *n*	
Pancreatic cancer	17
Gastric cancer	10
Gallbladder cancer	2
Bile duct cancer	2
Other malignancy	5
Choledocholithiasis	1
Reasons for EUS‐guided biliary drainage	
Surgically altered anatomy	20
Duodenal obstruction	10
Obscured ampulla due to invasive cancer	2
Segmental cholangitis difficult to control with ERCP	5
Site of the bile duct stenosis	
Distal bile duct	27
Hilar bile duct	6
Choledochojejunal anastomosis	2
Distal plus hilar	1
N/A	1
Puncture target	
B2	14
B3	23
Puncture site	
Stomach	30
Jejunum	7
Diameter of the target bile duct	4.5 (2.5–9.4)

*Notes*: Values are median (range), or *n*.

Abbreviations: ERCP, endoscopic retrograde cholangiopancreatography; EUS, endoscopic ultrasound; N/A, not applicable.

The results of the procedure are shown in Table 2. A total of 23 procedures were performed by Kei Yane, 12 by Masahiro Yoshida, and two by Takayuki Imagawa. Target bile duct puncture was successful in the 37 patients (100%). In one patient, the portal vein was punctured accidentally and recognized after guidewire insertion. Thus, the puncture needle was removed and the bile duct was punctured again. In six patients, bile duct puncture was successful and cholangiography and guidewire insertion could be performed, but the guidewire went into the peripheral bile duct and was difficult to advance to the hepatic hilum. For this reason, the guidewire was advanced to the hepatic hilum in two cases using the liver impaction technique.^19^ In the remaining four cases, the puncture needle was removed and re‐punctured to successfully insert the guidewire in the appropriate site. In these cases, the second puncture was easily performed with little change in the EUS image and dilated state of the bile duct. Finally, biliary access and guidewire placement to the target site were possible in all 37 patients. None of the patients required a 19‐gauge FNA needle for biliary access, and in all patients, biliary access was possible with a combination of a 22‐gauge FNA needle and a 0.018‐inch guidewire. In one case of segmental cholangitis with hilar biliary stenosis, in which B3 was punctured and a plastic stent (TYPE‐IT) was placed in the same area, the procedure was completed with a 0.018‐inch guidewire, whereas the other 36 cases were replaced with a 0.025‐inch guidewire. Fistula dilation was also successful in all the 37 patients. One patient scheduled for hepaticogastrostomy (HGS) with antegrade stenting underwent antegrade stenting alone owing to HGS stent breakage during stent deployment. Overall technical success was achieved in the 37 patients (100%). The procedures performed were EUS‐HGS with antegrade stenting (*n* = 18), EUS‐guided hepaticojejunostomy (EUS‐HJS) with antegrade stenting (*n* = 7), EUS‐HGS (*n* = 10), EUS‐guided antegrade stenting (*n* = 1), and EUS‐HGS with bridging stenting to the right liver lobe plus antegrade stenting (*n* = 1). Of these patients who only underwent antegrade stenting, HGS was performed with a plastic stent (TYPE‐IT) after antegrade stent placement. However, the stent was difficult to insert into the bile duct, and when an attempt was made to pull it back, the stent did not follow the delivery system and fell out of place, resulting in unsuccessful placement. The median procedure time was 35 min (16–125).

**TABLE 2:** Results of endoscopic ultrasound‐guided transhepatic biliary drainage using a 22‐gauge fine‐needle aspiration needle and a 0.018‐inch guidewire.

Success rate of biliary access	100 (37/37)
Success rate of fistula dilation	100 (37/37)
Dilation device	
Tapered tip ERCP catheter	7
Double‐lumen ERCP catheter	2
Tapered tip mechanical dilator	12
Balloon dilator	3
Drill dilator	13
Technical success rate	100 (37/37)
Stent type	
Plastic stent	7
Metal stent	6
Both	24
Clinical success rate	94.6 (35/37)
Procedure	
EUS‐HGS with AS	18
EUS‐HJS with AS	7
EUS‐HGS	10
EUS‐AS	1
EUS‐HGS with bridging stenting plus AS	1
Total procedure time, min	35 (16–125)
Adverse events	
Pancreatitis	1 (moderate)
Cholangitis	1
Cholecystitis	1
Stent dislodgement	1
Bleeding	0
Bile peritonitis	0

Abbreviations: AS, antegrade stenting; ERCP, endoscopic retrograde cholangiopancreatography; EUS, endoscopic ultrasound; EUS‐HGS, endoscopic ultrasound‐guided hepaticogastrostomy; EUS‐HJS, endoscopic ultrasound‐guided hepaticojejunostomy.

Clinical success was achieved in 35 patients (94.6%). Two patients did not show a sufficient reduction in the serum total bilirubin level after the procedure. Adverse events occurred in four (10.8%) patients. In the acute cholangitis patient owing to insufficient biliary drainage, HGS stent exchange and additional antegrade stent placement were performed via the HGS fistula site. In the acute cholecystitis patient, percutaneous transhepatic gallbladder aspiration was performed. In the patient with stent dislodgement 10 days postoperatively, computed tomography showed no obvious bile leakage, and the bile duct remained dilated. Therefore, EUS‐HGS with antegrade stenting was performed urgently, and further stent dysfunction was not observed. There was no clinically evident bile peritonitis, including 5 patients who required bile duct re‐puncture.

## DISCUSSION

Most previous studies on EUS‐guided transhepatic biliary drainage have reported that a 19‐gauge FNA needle is appropriate for puncturing the bile duct,^3^, ^7^, ^8^, ^12^ and the Japanese guidelines 2018 recommend the use of a 19‐gauge FNA needle.^13^ The guidelines also recommend the use of a 0.025‐inch or 0.035‐inch guidewire. This is because although the combination of a 22‐gauge FNA needle and a 0.021‐ or 0.018‐inch guidewire allows for bile duct puncture, subsequent device insertion is difficult.

However, target puncture is reportedly easier with a 22‐gauge FNA needle than with a 19‐gauge FNA needle on EUS‐FNA,^20^ and theoretically, the use of a 22‐gauge FNA needle could facilitate bile duct puncture on EUS‐HGS for the same reason. There have been reports of EUS‐HGS with a 22‐gauge FNA needle and a 0.018‐inch guidewire combination in difficult cases.^9^, ^15^ Although the previous 0.018‐inch guidewire was difficult to use owing to its poor maneuverability, good results have been reported with improvements in dilation devices mainly in high‐volume centers.^14^ Recently, reports of this technique have increased with an improved 0.018‐inch guidewire. Ogura et al. reported that puncture was possible in all cases, including non‐dilated bile ducts.^17^ These reports show that the combination of a 22‐gauge FNA needle and a 0.018‐inch guidewire makes puncture easier than the use of a 19‐gauge FNA needle, and is a useful rescue technique, particularly in difficult cases.

On the other hand, there have been no comprehensive reports on this technique's usefulness in non‐high‐volume centers during the procedure's induction phase. Thus, we conducted a retrospective observational study of the technical outcomes of all EUS‐guided transhepatic biliary drainage procedures with a 22‐gauge FNA needle and a 0.018‐inch guidewire, performed within a certain period of time at institutions during the procedure's induction phase, without limiting the target bile duct diameter.

The main advantage of a 22‐gauge FNA needle is its ease of biliary access. Experts in EUS‐guided transhepatic biliary drainage have reported that bile duct puncture with a 19‐gauge FNA needle is possible in most cases,^7^, ^8^ but is often difficult to reproduce by an endoscopist with less experience in EUS‐guided transhepatic biliary drainage.^10^ Although it is difficult to provide definitive data, we believe that a 22‐gauge FNA needle is not only easier to use for bile duct puncture but also makes it easier to advance the guidewire towards the hepatic hilum by selecting the appropriate puncture site, as it can puncture at a deeper angle than a 19‐gauge needle. From these points, we believe that the combination of a 22‐gauge FNA needle and a 0.018‐inch guidewire is highly advantageous in difficult and normal cases, particularly for endoscopists without extensive EUS‐guided transhepatic biliary drainage experience.

In this study, target bile ducts could be punctured in all patients. Moreover, guidewires could be advanced to the hepatic hilum by standard guidewire manipulation in 83.8% (31/37) of cases. Two cases required an advanced technique (liver impaction technique),^19^ and four cases required needle removal and re‐puncture owing to the difficulty of guidewire insertion to the hepatic hilum. The results compared favorably with previous reports.^10^, ^11^ In four cases that required re‐puncture, the second puncture was easily performed with little change on the EUS‐image and dilated state of the bile duct, possibly because of the less bile and contrast medium leakage.

A 0.018‐inch guidewire is considered to be difficult to use because of its poor maneuverability, and even after successful guidewire placement, fistula dilation is often difficult because of limitations in the applicable devices. However, 0.018‐inch guidewire‐compatible fistula dilatation devices have recently become available.^14^, ^15^, ^16^ In the present study, tapered tip ERCP catheters, mechanical dilators, balloon dilators, and drill dilators were used depending on each physician's discretion, and there were no unsuccessful cases during the fistula dilatation step.

This study has several limitations. First, the retrospective study design with a single‐center setting might cause patient selection bias. Also, there is no control group. Therefore, a multicenter validation study in a similar procedure induction situation should be performed. On the other hand, the number of included patients is relatively large compared with previous similar studies and hence is worth reporting.

Second, in most patients, we used an uncovered metal stent with a fine‐gauge (5.4 Fr) delivery system and a 7 Fr diameter dedicated plastic stent. In many patients, antegrade stenting was combined with EUS‐guided transhepatic biliary drainage. Therefore, it cannot be determined whether similar results would be achieved if EUS‐HGS with a thick delivery system metal stent was performed without antegrade stenting. However, in almost all procedures, cholangiography using an ERCP catheter and replacement with a 0.025‐inch guidewire were performed after fistula dilation. Therefore, theoretically, this method should be effective regardless of the details of subsequent procedures.

In conclusion, EUS‐guided transhepatic biliary drainage using a 22‐gauge FNA needle and a 0.018‐inch guidewire is a useful and promising option for endoscopists with limited EUS‐guided transhepatic biliary drainage experience in the procedure's induction phase in a non‐high‐volume center.

## CONFLICT OF INTEREST STATEMENT

None.

## Supporting information

Video S1 Endoscopic ultrasound‐guided hepaticojejunostomy using a 22‐gauge FNA needle and a 0.018‐inch guidewire.
